# Delivering Personalized Care at a Distance: How Telemedicine Can Foster Getting to Know the Patient as a Person

**DOI:** 10.3390/jpm11020137

**Published:** 2021-02-17

**Authors:** Janet D. Record, Roy C. Ziegelstein, Colleen Christmas, Cynthia S. Rand, Laura A. Hanyok

**Affiliations:** Department of Medicine, Johns Hopkins University School of Medicine, Baltimore, MD 21205, USA; rziegel2@jhmi.edu (R.C.Z.); cchristm@jhmi.edu (C.C.); crand@jhmi.edu (C.S.R.); lhanyok2@jhmi.edu (L.A.H.)

**Keywords:** telemedicine, person-centered care, communication, humanism

## Abstract

The promise of precision medicine is based on the use of new technologies to better characterize patients by defining individuals in the areas of genomics, proteomics, metabolomics and other aspects of biologic variability. Wise application of modern technology can similarly transform health visits with patients, allowing for better characterization of the patient’s individual life circumstances than possible in a traditional office visit. The use of, and experience with, telemedicine have increased significantly during the COVID-19 pandemic. Patients and clinicians report high satisfaction with telemedicine, and the quality of communication and patient-centeredness experienced in this setting are both rated highly. In this article, we explore the benefits offered by telemedicine in facilitating personalized care with particular focus on telemedicine delivered by video platforms. We propose strategies and skills specific to the effective implementation of personalized telemedicine, drawing on literature in patient-centered communication and home visits. While traditional in-person office visits continue to offer important opportunities such as thorough physical examination and the potential for enhanced non-verbal communication, telemedicine offers many important advantages that can facilitate the process of getting to know the patient as a person.

## 1. Introduction

The promise of precision medicine is based on the use of new technologies to better characterize patients by defining individuals in the areas of genomics, proteomics, metabolomics, and other aspects of biologic variability. Wise application of modern technology can similarly transform the patient–physician encounter, allowing for better characterization of the patient’s individual life circumstances than possible in a traditional office visit. 

Telemedicine presents challenges and opportunities for physicians and patients. Telemedicine has been defined as “the remote diagnosis and treatment of patients by means of telecommunications technology” [1]. Its use expanded dramatically in 2020 as clinicians and payors responded to the COVID-19 pandemic [2]. While this rapid adoption was necessitated by constraints imposed by the pandemic, many clinicians had limited time to learn best practices for providing patient-centered care via telemedicine. In this commentary, our objectives are to explore benefits offered by telemedicine in facilitating personalized care with particular focus on telemedicine delivered by video platforms; to propose strategies and skills specific to the effective implementation of personalized telemedicine, drawing on literature in patient-centered communication and home visits; and to advocate for an ongoing role for telemedicine beyond the pandemic that could provide an ideal patient-centered approach for outpatient medicine.

## 2. Personalized Medicine: Benefits Offered by Telemedicine

A previous article in this journal noted the distinction between “precision medicine” and “personalized medicine,” and recognized the importance of information derived from knowing the patient’s psychosocial situation to allow the tools of precision medicine to be tailored to the unique aspects of the patient as an individual [3]. Ziegelstein coined the term “personomics” to describe aspects of personalized medicine that derive from an understanding of the patients’ “personalities, health beliefs, social support networks, financial resources, and other unique life circumstances that have important effects on how and when a given health condition will manifest in that individual and how it will respond to treatment” [3]. Models of care delivery that involve clinician visits to patients’ homes have been shown not only to reduce short term costs of care, but also afford clinicians the opportunity to “obtain more-accurate information about the individuals than is possible when working in other settings, develop stronger relationships, and better calibrate the care plan to individual and caregiver needs” [4]. While some communication strategies can facilitate partial understanding of these aspects in an in-person office visit, and particularly by multiple office visits over many years, the ability to view the patient in the home setting through use of video visits offers opportunities for office-based physicians to better understand a patient’s environment even if a home visit is not feasible. 

In general, patient and clinician satisfaction with telemedicine is high [5,6,7,8,9,10,11,12,13,14,15,16,17,18]. While convenience factors, including travel-related time and cost savings, contribute to patient satisfaction [19], patients have also rated highly the quality of communication and patient-centeredness experienced in telemedicine visits [6,20]. For example, patients have reported high levels of satisfaction with feeling heard, having time to ask questions, being included in decision making, and feeling their concerns were addressed [6]. In fact, some patients have reported finding it easier to discuss difficult or personal topics over a video conference platform, as opposed to an in-person visit [21,22]. Some patients have reported improvement in self-efficacy in managing chronic conditions through telemedicine visits [21,23]. The personalized medicine benefits of telemedicine for patients and clinicians are shown in Table 1.

As an example, consider a 90 year-old woman with early dementia and hypertension who has recovered from surgery after a hip fracture two years ago. She lives with her daughter, who is also an older adult. For the patient and her daughter, telemedicine saves them the time and challenge of getting the patient to the office, especially as her mobility is limited. As the patient has access to a home blood pressure cuff, she is able to provide important data to the physician which may result in her feeling like a more active participant in her health care since she is actively monitoring these data herself. For her primary care physician, making telemedicine visits has allowed her to see the patient’s home and better assess home safety. For instance, the patient’s daughter has shown the physician around the home using the video camera, showing hallways, rugs, and assistive devices in the bathroom. The physician has seen how the patient’s medicines are organized in the home and offered suggestions for how to change pill bottle organization. These visits also provide opportunities for the physician to see photos of the patient’s loved ones that are present throughout her home. All of this occurred in an intimate yet time-efficient manner.

## 3. Patient-Centered Communication Skills and Telemedicine

Patient-centered communication has been linked to improved clinical outcomes, including patient-clinician relationships, diagnostic accuracy, patient understanding of medical information and recommendations, adherence to therapy, patient satisfaction with care, and physician wellbeing [24,25,26,27]. Patient-centered communication skills have received attention in academic medicine, both in in-person and telemedicine visits. The Accreditation Council for Graduate Medical Education (ACGME) has characterized competency in professionalism and communication skill domains as providing care that considers a patient’s unique needs and circumstances, incorporates perspectives of patients and caregivers, and anticipates ways to provide patient-centered care across transitions [28]. Further, in September 2020, the Association of American Medical Colleges (AAMC) released a set of telehealth competencies in the setting of the rapid increase in the use of video visits and remote patient care amid the COVID-19 pandemic. The AAMC Telehealth Competencies highlight the skills of building rapport effectively within video visits; involving patients’ social supports into visits when the patient provides consent to do so; and advocacy for improved access to telehealth while considering how a patient’s unique circumstances and “potential cultural, social, physical, cognitive, and linguistic/communication barriers to technology use” could impact a patient’s experience with telehealth [29].

Hashim has outlined aspects of patient-centered communication, noting a recommended sequence of eleven aspects of patient-centered medical interviewing that include introductions and building rapport, eliciting and defining the patient’s agenda, using open-ended and direct questions, and eliciting the patient’s perspective [30]. These patient-centered communication skills can be translated readily to the telemedicine setting (Table 2). Before or at the beginning of a visit, a patient can be invited to involve any caregiver or family member they wish in the visit, and this involvement may be facilitated by a telemedicine format, potentially even including a family member from a different location, or another member of the interprofessional care team. Opening a visit can involve brief non-medical conversation to build rapport and can focus on aspects of the patient’s life at home that may be visible or shared in the context of a video visit. Eliciting the patient’s perspective is a central component of patient-centered communication and applies to all aspects of a visit, including shared agenda-setting, history gathering, and medical decision making. When gathering history to explore the chief concern, asking a patient how their symptoms have affected their daily life is important, and a telemedicine visit from home may allow a patient to show, not just describe, how their life or function is affected by their illness. Nonverbal communication is as relevant in a telemedicine visit as in an in-person visit, and similar approaches apply. The clinician should convey attention focused on the patient, through use of eye contact and minimizing attention on note taking, other screens, or other information sources. The clinician can show evidence of active listening which can be conveyed through nodding and facial expressions that reflect empathy for the patient as they describe their symptoms or circumstances. Additional specific questions and phrases that clinically excellent physicians use to get to know a patient as a person [31] can be used just as easily in a telemedicine setting. Two examples that support personalized telemedicine and may leverage advantages offered through a video visit with a patient at home include, “Who are the people in your life who are most important to you?” and “What do you like to do in your free time?” The video visit can be used to “introduce” these people to the physician, by either involving that person or by showing a favorite photograph of that individual.

Opportunities to apply patient-centered communication strategies in telemedicine are relevant throughout any individual patient encounter. For example, consider caring for a middle-aged information technology consultant with Type 2 diabetes. When beginning the telemedicine encounter, the physician builds rapport by asking the patient about his recent experiences with work. The physician makes note of the Lego plastic construction toys in the background and learns that the patient loves building Legos with his daughter in his free time. After setting the agenda for the visit, data gathering includes the patient reporting recent home blood glucose readings and showing the physician the home exercise equipment he has been using, along with his exercise schedule. The physician works to minimize note-taking during the visit and makes use of touch typing to take some notes. Through the encounter, the physician uses some questions to get to know the patient as a person, learning about the importance of the patient’s wife to him and her role supporting him in self-managing his diabetes. 

The importance of being fully present with the patient in an interaction is an important aspect of getting to know patients as individuals [32]. Prince-Paul and Kelley have described the features of being present as part of “mindful communication” which they define as being an active process where both clinician and patient are “attentive to the timing, nature, and context of the dialogue exchange” [33]. They note that this type of communication, “helps direct care that is patient-centered, reflective, and relational.” Zulman and colleagues offer five suggestions to facilitate presence and connection in any clinician–patient encounter, which overlap with the patient-centered communication strategies presented above: prepare with intention; listen intently and completely; agree on what matters most; connect with the patient’s story; and explore emotional cues [32]. Being fully present with the patient in a telehealth visit requires certain practices that are different from those used in the in-person visit. There should be attention to the lighting, sound, and items present in the field of view. If possible, telephones should be silenced. The clinician and patient should aim to focus on the visit and should avoid multi-tasking. Nguyen has noted the importance of establishing realistic expectations before the visit to enhance patient satisfaction with telemedicine [5]. The patient should be asked to find a comfortable and confidential space for most of the visit; however, if able, the clinician should set the expectation that the patient be able to walk from room to room with the video on to show the clinician aspects of the home during the visit or engage others in the discussion as they choose.

## 4. Getting to Know the Patient at Home: Adapting Components of Home Health Visits to Inform Personalized Telemedicine

When clinicians provide medical care through home visits, this provides vastly more information about a patient’s environment, can enhance the patient–physician relationship, and allows for more personalized medical care [4]. Unfortunately, in usual medical practice, home visits are challenging and occur only rarely. House calls—once quite common in general medical practice—are exceptionally rare [34]. Fewer than 1% of elderly Medicare patients in the U.S., even those who may be frail, homebound, and near the end of life, receive home visits [35]. While there is some evidence that house calls are more frequent in recent years [36], fewer physicians are making those visits [37]. The value of home visits is recognized in family medicine and pediatric residency programs but is not a program requirement in the U.S. and is relatively infrequent in a resident’s experience [38,39]. Training in how to provide home visits is a core requirement of geriatric fellowship training programs [40], but this applies to a small fraction of medical trainees overall. Recognizing that the training of future physicians often seemed to miss opportunities for addressing patient-centered care, a patient-centered care curriculum was developed at Johns Hopkins University School of Medicine in 2007 [41,42,43,44,45]. One component of this curriculum is a home visit after a patient is discharged from home, with goals of learning how to improve planning for patient-centered transitions of care [43].

Telemedicine provides an opportunity for physicians to make virtual house calls [46,47,48,49,50], and—like a home visit—may be, “a unique opportunity for the physician to learn about social and functional elements of the patient’s day-to-day life that influence medical care and outcomes” [43]. A telemedicine house call can help clinicians to better understand the patient and support systems. With careful observation and following certain guidelines, a virtual home visit can provide insight into the patient’s life that may be similar to what can be obtained from an actual home visit, providing an understanding of the patient as a person that, “can inform medical decision-making, challenge assumptions and highlight implicit biases, and deepen the human connection between physician and patient” [43]. Virtual house calls through telemedicine have been described as effective for geriatric patient care, even for those who have cognitive impairment or dementia, allowing for provision of patient-centered care following the Geriatric 5 M framework [51]: Mind, Mobility, Medications, Multicomplexity, and what Matters Most [46]. 

Telemedicine visits that adapt components of home visits can expand the possibilities for the agenda of a visit [52]; see Table 3. The INHOMESS mnemonic [34] for components of a comprehensive in-person home visit provides a helpful frame for a personalized telemedicine visit. The INHOMESS checklist, developed with needs of homebound patients in mind, includes assessments around impairments/immobility, nutrition, home environment, other people, medications, examination, safety, spiritual health, and services [34]. Rather than just describing in an office visit, a patient can demonstrate their home environment, and the physician can simultaneously assess aspects of home safety while getting to know the patient as a person. The clinician can inquire about important aspects of the patient’s home situation and observe where the patient eats, watches television, reads, or exercises. The clinician can view the patient’s favorite rooms in the home setting, where the patient spends the most time, where they sleep, eat, use the toilet, shower, or bathe. The patient can introduce the clinician to other members of the family or to pets. The structure of the home can be assessed, including whether it is more than one level, how many stairs must be climbed, the distance from the bedroom to the bathroom, whether there are carpets, oxygen tubing, electrical cords or inadequate lighting that might lead to falls. A patient can demonstrate how they navigate daily activities at home, such as navigating stairs or preparing food. With a patient’s permission, the clinician can view the types of food in the home, assessing for availability of food that supports a patient’s self-management of any conditions they have. The patient can be asked to show the clinician how medications are stored and organized to better understand challenges with adherence and obtain a visual impression of regimen complexity. When the visit involves a caregiver or family member to help manage the camera, provide information, or assist in demonstrations the physician can observe the relationship and interaction between a caregiver and patient. This may provide insight into whether there is adequate caregiving support or caregiver burnout. Other aspects of home safety can be assessed, including presence of fire extinguishers and smoke detectors. Table 3 summarizes a comparison of benefits of telemedicine and office visits.

## 5. Limitations of Telemedicine to Provide Personalized Care

Telemedicine is surely not a panacea (Table 4). In-person visits can facilitate patient–clinician relationship building in important ways, in part because office visits allow for effective non-verbal communication and facilitate social connection. Some domains of the physical examination are limited in a telemedicine visit. If serious, acute illness is suspected, in-person emergency medical care would be recommended. However, patients, with or without a caregiver, may be able to engage in a range of maneuvers that can facilitate neurologic, musculoskeletal, dermatologic, and other examination components, even jugular venous pressure assessment [19,50,53]. Remote monitoring technologies, which tend to be underutilized, can be leveraged to provide additional clinical data and empower patients as active participants in self-monitoring, and telemedicine visits provide an opportunity for any teaching that may be needed to ensure patients are using these devices correctly. Interprofessional home care teams, with home visits by nursing or other professionals, can help inform physical assessment for telemedicine visits. 

Another potential limitation of telemedicine relates to access to required technology. A recent study found that early in the COVID-19 pandemic, there were inequities in access to telemedicine visits, and that video visits specifically were less likely to have taken place for older patients, female patients, racial and ethnic minorities, and poorer patients [54]. A significant proportion of the population lacks access to a primary care provider for reasons such as living in a geographically remote location or local shortage of physicians [5]; telemedicine’s ability to provide some of this access has been highlighted [55] but is unlikely to meet all needs. For example, in the United States, while most households have a computer [56], the Pew Research Organization found that 10% of American adults do not use the Internet [57]. High speed internet access, required for telemedicine video visits, is also less available in rural areas [58]. Some individuals may have access to telephone visits instead of video encounters; however, telephone visits have significant limitations. Not only is the ability to understand the patient’s home environment and conduct a physical examination markedly reduced with telephone-only visits, some individuals are also unable to utilize telephones because of sensory impairments or lack of phones. Approaches beyond telemedicine will be needed in these instances. Table 4 summarizes potential limitations of telemedicine, paired with potential solutions and examples from the authors’ practices. 

## 6. Conclusions

The demand and constraints of the COVID-19 pandemic have created the unique circumstance where many physicians have had to become familiar with telemedicine for routine patient care visits. While the learning curve has been rapid and not without challenges, telemedicine can provide important opportunities for physicians to learn more about their patients within their real-life environments, including their physical, social, and emotional resources and barriers. 

The precision medicine toolkit includes many new technologies to better characterize patients through genomics, proteomics, metabolomics and other “-omics” that define biologic variability. Telemedicine incorporates new communications technologies to allow for similarly informative characterization of patients’ individual life circumstances to tailor diagnostic and treatment strategies. While traditional in-person office visits continue to offer important opportunities such as thorough physical examination and the potential for enhanced non-verbal communication, telemedicine offers many important advantages that can facilitate the process of getting to know the patient as a person. The ideal approach to personalized care may a combination of in-person and telemedicine visits.

## Figures and Tables

**Table 1:** Telemedicine—personalized medicine benefits for patients and clinicians.

Benefits for Patients	Benefits for Clinicians
No travel required Time saved; less time away from work/school/home/caregiving/other Cost saved More convenient, especially for patients for whom mobility is challenging More readily able to involve other informants and sources of support	No travel required Opportunity to work from home when doing telemedicine, saving travel time
Can be done anywhere there is internet/phone connection within licensing limits of the physician Home environment usually a more comfortable setting for the patient	Opportunity to learn about patient’s home environment Note any family and/or support in home, and obtain medical history from family or caregivers Window into what is important to the patient Safety of environment (lighting, risk factors for falls, presence of stairs, etc.)
May increase access to medical care despite geographically remote locations and/or shortage of physicians in area	Opportunity to review medication organization and reconcile discrepancies, nutrition patterns in home environment
Reduce risk of exposure to infection in waiting room and office	Reduce risk of exposure to infection
When feeling known as a person, potential for increased trust in clinician and satisfaction with medical care	Potential for increased sense of satisfaction with work when learning more about patient as a person

**Table 2:** Strategies to provide personalized telemedicine: patient-centered communication skills.

Strategies to Provide Personalized Telemedicine: Patient-Centered Communication Skills	
Planning participants	Ask patient whether they would like to involve a family member, caregiver, or other person in their support network in the telemedicine visit.
Opening the visit	Begin with introductions if the patient is involving another person in the visit. Build rapport by opening with brief non-medical conversation that could explore patient’s life at home.
Elicit the patient’s perspective	Shared agenda-setting History gathering: Prompt the patient to describe how their symptoms have impacted their daily life and consider an opportunity to use the video visit to share visual examples of this, where applicable. Shared decision-making
Show evidence of active listening	Be fully present. Minimize distractions in the environment. Use nonverbal communication such as nodding and facial expressions that convey empathy. Notice and follow up on patient’s emotional cues.

**Table 3:** Comparison of the benefits of in-person office visits compared to telemedicine visits.

Domain	Office Visit	Telemedicine Visit
Assessing patient’s self-management	Patient or caregiver report; review medication adherence by memory or list	Patient can show their organization system; can demonstrate use of inhalers or other medical devices
Involving family/caregivers	If a family member/caregiver accompanies patient to office, they may participate in the office visit or stay in waiting room. Family members living far away may not be able to participate.	Potential to involve more care partners in more visits, even individuals at distant sites.
Home environment	Limited, indirect information about level of organization in home, safety of home environment	Potential for visualizing much of the home environment for level of organization and safety
Coordination of care and co-management	Single physician–patient dyad at one visit	Potential for more than one physician on patient’s care team to join all or part of the same telemedicine visit (e.g., primary care provider and specialist)
Time and convenience	Time and convenience favors physician/care team	Potential for favorable time and convenience benefits for both physician and patient, when the patient is comfortable with the telemedicine modality and has access
Physical examination	Opportunity for complete, in-person examination	Limited visual assessment; some maneuvers may be assessed with involvement of patient and caregiver
Nonverbal communication	Can be facilitated by in-person environment	Can be limited somewhat by video conference modality
Patient Access	Transportation, and insurance coverage vs. sliding scale payment model	Requires access to a device and internet connection

**Table 4:** Potential limitations of telemedicine and potential solutions.

Potential Limitation of Telemedicine	Potential Solutions	Examples from the Authors’ Practices
Potential challenges in building the patient–clinician relationship, due to physical distance and related barriers to non-verbal communication	Use strategies as above to build rapport through non-medical conversation that explores patient’s life at home and refers to the setting visible in the patient’s environment during telemedicine visit. Select an in-person office visit as the modality for some future appointments if feasible.	A 65-year-old man was seen for an initial telemedicine visit and began noting, “I can see that a virtual visit might be alright if I had a very specific issue but how can this be good for a general appointment?” The clinician spent the first few minutes of the visit asking general questions about the patient’s life [31] while taking advantage of the video home visit to ask the patient the walk around the house and illustrate certain things in the narrative (e.g., his home office, his dog, his woodwork). This helped the patient feel that the clinician was interested in knowing him as a person. At the end of the visit, the patient commented, “I really felt like you got to know me better than on any of my in-person visits with other doctors.”
Physical examination limited	Engage a caregiver or family member in assisting with some maneuvers, such as demonstrations of motor strength, gait, and musculoskeletal examination. Leverage use of remote monitoring technologies, such as a blood pressure monitoring device, thermometer, pulse oximeter, scale, glucometer, and smartphone-enabled personal electrocardiogram. Physical assessments by members of interprofessional home care teams, with home visits by a nurse, physical therapist, occupational therapist, or other team member	An 86-year-old woman with heart failure and chronic obstructive pulmonary disease noted mild dyspnea during a follow-up telemedicine visit. She noted no orthopnea or paroxysmal nocturnal dyspnea. The remote video-assisted physical examination showed no lower extremity edema and examination of the jugular veins showed no distention [53], making volume overload unlikely and guiding the appropriate treatment.
Potential for limited access to telemedicine, due to financial barriers, geographic barriers, lack of familiarity or access to technology, or other reasons	Telephone visits In-person office visits Home visits	A 59-year-old woman lives in an area without good internet connection. Attempts at videoconferencing proved challenging, so visits were by telephone. The patient reported palpitations, and the history suggested that this symptom was related to anxiety and stress, exacerbated recently by the pandemic and national political events. The clinician recommended that she purchase a smartphone-enabled personal electrocardiogram device and then obtain and send recordings of heart rhythm during episodes of palpitations. These all showed normal sinus rhythm. Subsequent telephone visits were spent discussing stress-reducing techniques which eliminated further episodes.

## Data Availability

This work does not report any data except that already published and referenced.
